# Cooperative Anti-Diabetic Effects of Deoxynojirimycin-Polysaccharide by Inhibiting Glucose Absorption and Modulating Glucose Metabolism in Streptozotocin-Induced Diabetic Mice

**DOI:** 10.1371/journal.pone.0065892

**Published:** 2013-06-06

**Authors:** You-Gui Li, Dong-Feng Ji, Shi Zhong, Zhi-Qiang Lv, Tian-Bao Lin

**Affiliations:** Sericultural Research Institute, Zhejiang Academy of Agricultural Science, Hangzhou, China; Universidade Federal do Rio de Janeiro, Brazil

## Abstract

We had previously shown that deoxynojirimycin-polysaccharide mixture (DPM) not only decreased blood glucose but also reversed the damage to pancreatic β-cells in diabetic mice, and that the anti-hyperglycemic efficacy of this combination was better than that of 1-deoxynojirimycin (DNJ) or polysachharide alone. However, the mechanisms behind these effects were not fully understood. The present study aimed to evaluate the therapeutic effects of DPM on streptozotocin (STZ)-induced diabetic symptoms and their potential mechanisms. Diabetic mice were treated with DPM (150 mg/kg body weight) for 90 days and continued to be fed without DPM for an additional 30 days. Strikingly, decrease of blood glucose levels was observed in all DPM treated diabetic mice, which persisted 30 days after cessation of DPM administration. Significant decrease of glycosylated hemoglobin and hepatic pyruvate concentrations, along with marked increase of serum insulin and hepatic glycogen levels were detected in DPM treated diabetic mice. Results of a labeled ^13^C_6_-glucose uptake assay indicated that DPM can restrain glucose absorption. Additionally, DPM down-regulated the mRNA and protein expression of jejunal Na^+^/glucose cotransporter, Na^+^/K^+^-ATPase and glucose transporter 2, and enhanced the activities as well as mRNA and protein levels of hepatic glycolysis enzymes (glucokinase, phosphofructokinase, private kinase and pyruvate decarboxylas E1). Activity and expression of hepatic gluconeogenesis enzymes (phosphoenolpyruvate carboxykinase and glucose-6-phosphatase) were also found to be attenuated in diabetic mice treated with DPM. Purified enzyme activity assays verified that the increased activities of glucose glycolysis enzymes resulted not from their direct activation, but from the relative increase in protein expression. Importantly, our histopathological observations support the results of our biochemical analyses and validate the protective effects of DPM on STZ-induced damage to the pancreas. Thus, DPM has significant potential as a therapeutic agent against diabetes.

## Introduction

Diabetes mellitus is characterized by chronic hyperglycemia with disturbances of carbohydrate, fat and protein metabolism that result from deficiency of insulin secretion and/or insulin resistance [1]. Streptozotocin (STZ) is widely used to induce diabetes-like symptoms such as polydipsia, polyphagia, polyuria and hyperglycemia in experimental animals [2]. It is well known that carbohydrates in the diet are hydrolyzed into monosaccharides, and then absorbed through the intestine by a transepithelial transport system. A number of studies have reported that glucose absorption increases following enhanced activity, mRNA, and protein levels of intestinal SGLT1, Na^+^/K^+^-ATPase and GLUT2 in the brush border membrane (BBM) and basolateral membrane (BLM) of STZ-induced diabetic animals [3]–[6]. Sustained supra-physiological glucose level resulting from excessive glucose assimilation may be toxic to β-cells, leading to deterioration of insulin control [7]. Continuous deterioration of endocrine control exacerbates metabolic disturbances by altering the activities of glycolysis and gluconeogenesis enzymes, leading to impairment of peripheral glucose utilization and augmentation of hepatic glucose production [8], [9]. Therefore, inhibition of glucose absorption in the small intestine, regulation of key enzymes involved in carbohydrate metabolism in the liver, and repair of destroyed β-cells in the pancreas seem to be useful strategies to lower blood glucose levels in individuals with diabetes.

Due to their comparatively fewer side-effects and lower costs, phytochemicals derived from natural resources are important alternatives for the treatment of various diseases, including diabetes. In China and other Asian countries, mulberry leaves and their extracts are used as alternative treatment for diabetes [10], [11]. In recent years, 1-deoxynojirimycin (DNJ), which is found in mulberry trees, has attracted considerable interest because of its effective and specific inhibition of various carbohydrate-degrading enzymes involved in a wide range of important biological processes, such as intestinal digestion, hepatic glycogen breakdown, lysosomal catabolism of glycoconjugates, and maturation of the sugar chains in glycoproteins [12], [13]. In our previous studies, we showed that DNJ can significantly reduce blood glucose concentration after a meal [14], [15]. And ample evidence has established that polysaccharides have anti-diabetic effects [16], [17], our previous studies also reported that the polysaccharide (P) from mulberry is useful in protecting against alloxan-induced pancreatic islets damage *in vivo*. Furthermore, we showed that the antihyperglycemic efficacy of the combination of DNJ and polysaccharide from mulberry was better than that of either of them alone [15]. However, the mechanisms behind these effects are not yet fully understood. One possible explanation is that the combination of DNJ and polysaccharide from mulberry leaves not only decreases blood glucose, but also ameliorates pancreatic and β-cell functions in chronic diabetics. The purpose of this study was to further evaluate the following: [(1)] the therapeutic effects of DNJ-polysaccharide mixture (DPM) on STZ-induced diabetic mice, [(2)] changes in blood glucose levels after cessation of DPM intake, [(3)] the mechanisms of anti-diabetic effects of DPM.

## Materials and Methods

### Preparation of DNJ and polysaccharides

DNJ and polysaccharides were extracted from Mulberry (*Morus Multicaulis* Perr.) leaves. DNJ was purified by LC-MS systems (Waters, USA) and polysaccharides were purified using HPLC (Waters, USA) as described by Li et al. (2011) [15]. Purity of both DNJ and polysaccharides was more than 95% based on HPLC analysis.

### Experimental design

Experiments were performed with male ICR mice (25±2 g) that received a standard diet with free access to tap water. After one week of acclimatization, weight-matched mice were subjected to a 16-hour fast. Diabetes was induced as previously described [18] with some modification. Briefly, streptozotocin (STZ) (Sigma-Aldrich) (125 mg/kg b.w.) dissolved in 0.1 M cold citrate buffer (pH 4.5) was injected intraperitoneally on days 1 and 4. Seven days after STZ treatment, mice with 12 h fasted blood glucose concentration between 15–20, 20–26, 26–31 mmol/l were selected for the experiment. Normal mice and those with different degrees of STZ-induced diabetes were divided into seven groups (n = 20 per group) as follows: Group I: normal mice receiving 0.9% saline; Group II: STZ-induced diabetic mice (26–31 mmol/l blood glucose) receiving 0.9% saline; Group III: STZ-induced diabetic mice [(26]–[31)] administered with 50 mg/kg b.w./d of DNJ + 100 mg/kg b.w./d of polysaccharide (DPM); Group IV: STZ-induced diabetic mice [(20]–[26)] receiving 0.9% saline; Group V: STZ-induced diabetic mice [(20]–[26)] administered with DPM (150 mg/kg b.w./d); Group VI: STZ-induced diabetic mice [(15]–[20)] receiving 0.9% saline; Group VII: STZ-induced diabetic mice [(15]–[20)] administered with DPM (150 mg/kg b.w./d). All samples were dissolved in 0.9% saline and administered intragastrically for 90 days, and the mice were continued to be fed normally without DPM for an additional 30 days. The 150 mg/kg dosage of DPM was selected based on our previous studies on its protective effect against alloxan-induced injury in diabetic mice [15]. During the experimental period, blood glucose and body weight were determined (in a random set of 10 mice per group, fasted 12 h) at 0, 30, 60, 90, 105 and 120 days. Oral glucose (sucrose) tolerance test (OG(S)TT) was carried out at 90 days in overnight fasted mice (group I, VI, VII), administered with equivoluminal 0.9% saline and DPM (150 mg/kg b.w.) 15 min prior to administration of 30% glucose (sucrose) solution (3 g/kg) to each group (n = 10). Glucose concentration was measured in peripheral blood taken from the tail vein at 0, 30, 60, 90 and 120 min. At the end of OGTT, animals (n = 10) were sacrificed, and their serum and liver were immediately collected for biochemical estimations. BBM (for SGLT1) and BLM (for Na^+^/K^+^-ATPase and GLUT2) were isolated from the jejunum as described by Boyer et al. [19]. BBM, BLM and liver of the animals were frozen in liquid nitrogen for subsequent isolation of RNA and proteins. In addition, for the assessment of glucose absorption and transport in intestine, DPM (150 mg/kg b.w.) and labeled ^13^C_6_-glucose (1 g/kg b.w.) were administered intragastrically with 15-minute intervals into fasting mice (groups I, VI, VII; n = 5) at 90 days. Blood and small intestine samples were collected 30 min later and processed for GC/MS analysis as previously described [20]. All animal experiments were carried out following protocols approved by the Institutional Animal Care and Use Committee of the Zhejiang Academy of Agricultural Science.

### Biochemical estimations

Glycosylated hemoglobin and insulin levels in the serum, and glycogen and pyruvate levels in the liver were determined using kits purchased from R&D Systems, USA and SuZhou Keming Bioengineer Company, China, respectively, following the manufacturer's instructions. GK [21], PFK [22], PK [23], PEPCK [24] and G-6-Pase [25] activities were assayed as described previously.

### Quantitative real-time reverse transcription-PCR analysis

Total RNA was extracted using TRIZOL reagent according to the supplier's instructions (Hangzhou biosci biotech co., ltd, China). Reverse-transcription was performed using RevertAid^TM^ First-Strand cDNA Synthesis Kit for RT-PCR as previously described [15]. Primer sequences are available on request.

### Western blot analysis

Samples were suspended in lysis buffer (50 mM Tris (pH 8.0), 150 mM NaCl, 0.1% SDS, 0.5% sodium deoxycholate, 1% NP40, phenylmethylsulfonyl fluoride at 100 µg/mL, aprotinin at 2 µg/mL, pepstatin at 1 µg/mL, and leupeptin at 10 µg/mL), and placed on ice for 30 min. After centrifugation at 15,000 g for 15 min at 4°C, the suspension was solubilized with SDS-stopping solution (4% SDS, 2 mM EDTA, 8% β-mercaptoethanol and 50 mM Tris; pH 6.8) and total protein was measured using the bicinchoninic acid assay (SuZhou Keming Bioengineer Company, China) following the manufacturer's recommendations. Samples (50 µg of protein) were separated by SDS-PAGE using 10% gels. The proteins were transferred to nitrocellulose membrane using 400 mA current (3 h; 4°C). The membranes were blocked with 5% skim milk (1 h), followed by a second blockage (1 h) with 2.5% gelatin. Primary antibodies (anti-GK-ab37796, -PFK-ab119796, -PK-ab38240, -PDK2-ab92959, -PEPCK-ab70358, -Na^+^/K^+^-ATPase-ab110730, -SGLT1-ab652 and -GLUT2-ab54460) were obtained from Abcam (Britain). Incubation period was 2 h (room temperature) for the detection of all forms of MAPKs except the phosphorylated forms, for which it was 12 h (4°C). All steps of blocking and incubation were followed by washing (every 5 min, 3 times) of the membranes with TBST (Tris 10 mM, NaCl 150 mM, Tween-20, 0.05%; pH 7.5). The blots were developed using an enhanced chemiluminescent (ECL) kit (Amersham).

### Histopathological examination

After 90 days of treatment with DPM (150 mg/kg b.w./d), the pancreas and livers of three animals from each group (I, VI, VII) were separated and stored in 10% formalin. They were later sectioned using a microtome, dehydrated in graded alcohol, embedded in paraffin, stained with haematoxylin and eosin, and examined using a Lecia-DM2500 (Germany) microscope.

### Effect of DPM on the activities of GK, PFK and PK *in vitro*

The standards of GK, PFK and PK enzymes were purchased from Sigma-Aldrich (St. Louis, MO, USA). Their activities were measured as described in previous studies [21]–[23]. DPM at final concentrations of 0, 1.25, 2.5, 5, 10, 20, 40 µg/ml were incubated with GK, PFK, PK enzymes for 15 min before the assays.

### Protective effect of DNJ and polysaccharide against STZ-induced pancreaticβ-cell damage

The pancreatic β-cell line INS-1 obtained from the Institute of Biochemistry and Cell Biology, Chinese Academy of Sciences was cultured according to the manufacturer's instructions. Cells were plated at a density of 1×10[5] cells/well on 96-well plates for viability assay. After 24 h, STZ solution (final concentration 6 mM) was added to each well and the cells were exposed to STZ for 24 h or kept untreated as controls. The STZ dosage (6 mM) was selected based on previous reports [26]. Meanwhile, the cells were incubated for 24 h in the presence or absence of DNJ and polysaccharide (P) (dissolved in RPMI-1640 to a final concentration of 200 µg/ml). After cultivation for 24 h at 37°C in a humidified 5% CO_2_ incubator, morphological characteristics of β-cells were recorded with an inverted phase contrast microscope (Leica, Germany). Viability of the cells was determined by an MTT assay, reading absorbance at 570 nm with a Benchmark microplate reader (Bio-Rad, California).

### Statistical analysis

Results are reported as mean ± S.D. ANOVA was used to evaluate the difference between multiple groups. If significance was observed between groups, a Duncan's multiple range test (DMRT) was conducted to compare the means of two specific groups using a commercially available statistical software package (SPSS for Windows, V. 12.0, Chicago, USA), with *P*<0.05 considered statistically significant.

## Results

### DPM has therapeutic effects on STZ-induced diabetic mice

**Figure 1:**
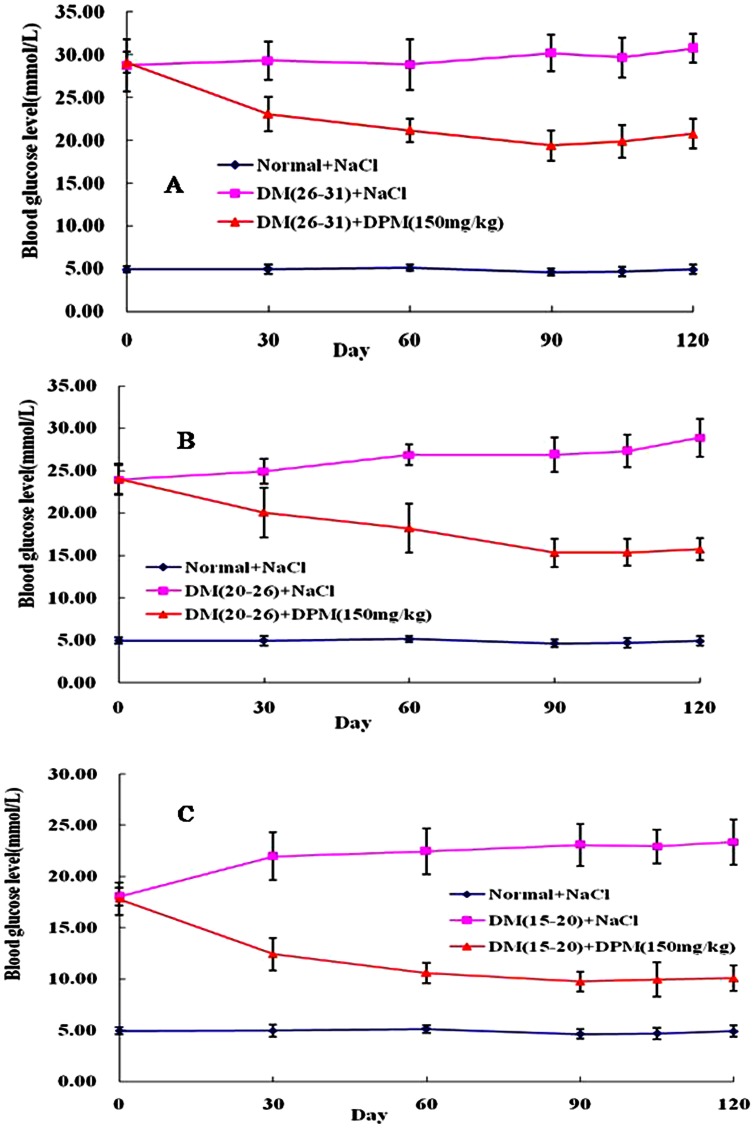
Effect of DPM (150 mg/kg) on the level of blood glucose in STZ-induced diabetic mice after an overnight fast (12 h). The blood glucose level was determined from the tail vein. Each point represents the mean ± SD for 10 animals in each group.

**Figure 2:**
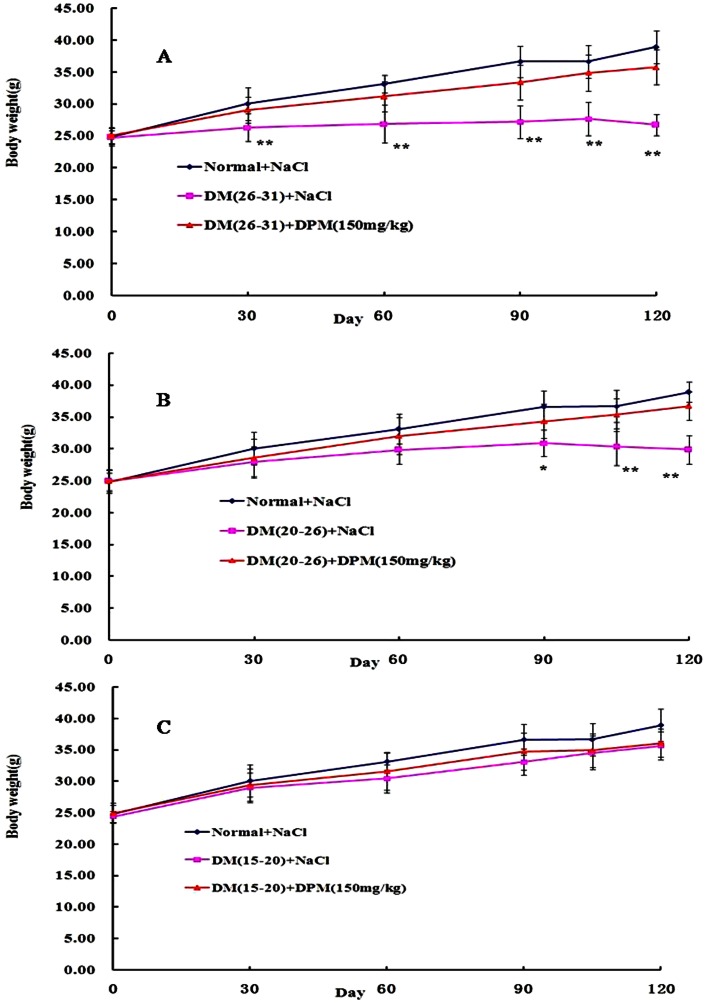
Effect of DPM (150 mg/kg) on the body weight in STZ-induced diabetic mice. Each point represents the mean ± SD for 10 animals in each group. *P<0.05, **P<0.01vs. Normal.

In order to identify the therapeutic effects of oral administration of DPM (150 mg/kg) on STZ-induced diabetes, mice were fasted for 12 h before determining their blood glucose levels. As showed in Figure 1, there was a remarkable (P<0.05) elevation in the levels of blood glucose of STZ-induced diabetic mice compared with control animals. Upon treatment with DPM (150 mg/kg) for 90 days, the blood glucose levels of all diabetic mice with different degrees of injury induced by STZ were markedly diminished (29.09±1.24, 24.01±1.82, 17.82±1.57 to 19.38±1.16, 15.29±1.68, 9.75±0.96 mmol/L, respectively). Importantly, the decrease in blood glucose level was not reversed in lower hyperglycemia mice groups (V, VII) after stopping DPM administration for another 30 days (Fig. 1B, C). A slight increase in blood glucose was observed in the more severe hyperglycemia group (III) after stopping DPM administration (Fig. 1A), but did not reach statistical significance compared to the blood glucose levels measured at the 90^th^ day of DPM administration. Additionally, striking decrease of glycosylated hemoglobin (HbA1c) indicated the decrease of glucose concentration in blood, while significantly increased serum insulin indicated that the damaged pancreas had been ameliorated in DPM treated diabetic mice (Table 1). Furthermore, the body weights of normal mice increased throughout the 120 days of observation (Fig. 2). Although slight increase in the body weights of the mice in Group II and Group IV were detected during the experiment, they were still significantly lower compared to the control animals. However, the body weights of mice in Group VI were not markedly different from that of the control group. The body weights of all animals in the DPM treatment groups increased gradually with the passage of time, and no significant difference was observed among these groups compared to the control group. Moreover, no case of diarrhea was found in DPM treated mice throughout the experiment. Taken together, these results indicate that DPM has therapeutic effects against diabetes.

**Table 1:** Levels of serum HbA1c and Insulin in normal and experimental groups of mice. (Mean ±S.D., n = 10).

Groups	HbA1c (%)	Insulin (mU/ml)
Normal control	5.46±0.26	20.58±0.65
Diabetic control	11.17±0.65^**^	13.89±0.71^**^
Diabetic+DPM (150 mg/kg)	8.25±0.47^**,##^	16.29±0.55^**,##^

Data are expressed as mean ± S.D., n = 10. *P<0.05, **P<0.01, compared with normal control;

# *p*<0.05, ## *p*<0.01 compared with diabetic control.

### DPM inhibits disaccharide decomposition and glucose absorption

**Figure 3:**
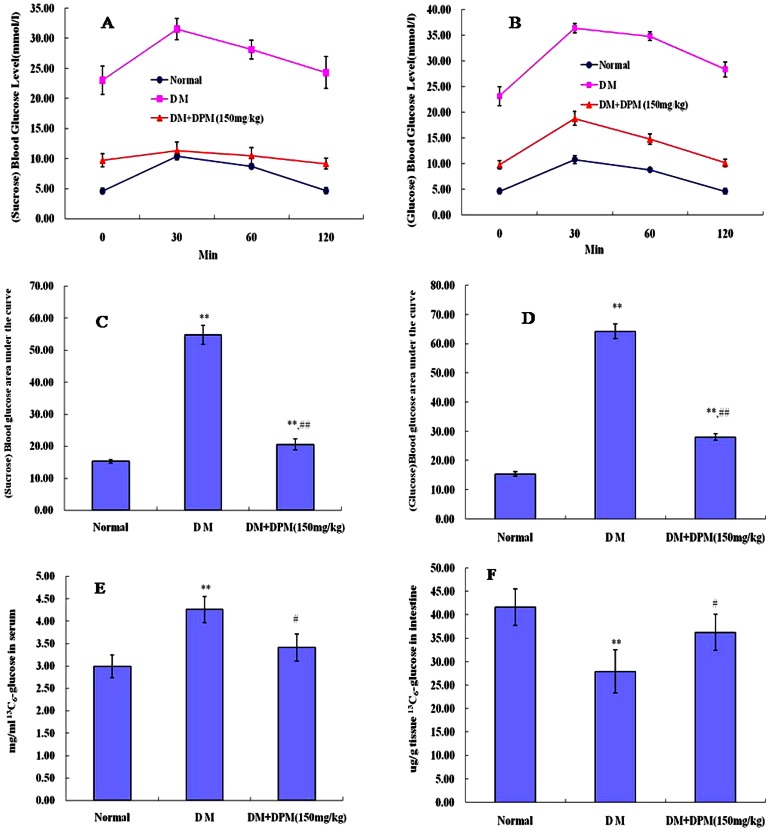
Oral sucrose tolerance tests (OSTT), oral glucose tolerance tests (OGTT) and labeled ^13^C_6_-glucose uptake assay. Results represent OSTT (A) and the corresponding calculated relative area under the curve (AUC) for glucose concentration (C). OGTT results are shown in (C) and its corresponding AUC for glucose concentration shown in (D). ^13^C_6_-glucose concentrations in serum and intestine are shown in E and F, respectively. Results are expressed as means ± SD (A-D, n = 10; E and F, n = 5 per group). *P<0.05 and **P<0.01 vs control groups.

In order to further study the anti-diabetic effects of DPM, oral sucrose and glucose tolerance tests (SGTT, OGTT) were performed on mild fasting hyperglycemic mice (groups VI, VII) after 90 days of treatment. Both sucrose and glucose treatment resulted in impairment of glucose tolerance as revealed by the glucose tolerance curve (Fig. 3A, B) and the calculated relative area under the curve (AUC) for glucose concentration (Fig. 3C, D). The maximal blood glucose levels were 31.53±1.77 and 36.40±0.87 mmol/l at 30 min after sucrose and glucose administration, respectively. Strikingly, pretreatment of animals with DPM (150 mg/kg) showed significant preventive effects against hyperglycemia induced by sucrose. The blood glucose levels were significantly lower in pretreated animals than in diabetic controls (P<0.01), and showed no significant fluctuation during the 2 h experiment (Fig. 3A). These results indicate that DPM is a particularly potent inhibitor of intestinal sucrase, capable of inhibiting disaccharide digestion and thus delaying its absorption. Similarly, we observed remarkable amelioration of glucose tolerance in OGTT, as shown in the glucose tolerance curve (Fig. 3B) and the AUC for glucose concentration (Fig. 3D). The increase in blood glucose level from 0 min to 30 min in DPM treated diabetic mice (9.03±1.31 mmol/l) was significantly lower than that in the corresponding control (13.25±1.78 mmol/l), indicating that DPM is capable of inhibiting glucose absorption. To verify this conclusion, we next took advantage of labeled ^13^C_6_-glucose to trace glucose absorption and transport in the intestine. As seen in Fig. 3E, the blood ^13^C_6_-glucose level was remarkably lower in DPM treated mice than in diabetic controls (3.41±0.30 vs. 4.26±0.29 mg/ml, P<0.05), but the result was opposite in the small intestines of the respective animals (36.28±3.88 vs. 27.96±4.60 ug/g tissue, P<0.05) (Fig 3F). Together, these results indicate that DPM can not only inhibit digestion of disaccharides to monosaccharides but also inhibit glucose absorption, resulting in significant reduction in the blood glucose concentration after a meal.

**Figure 4:**
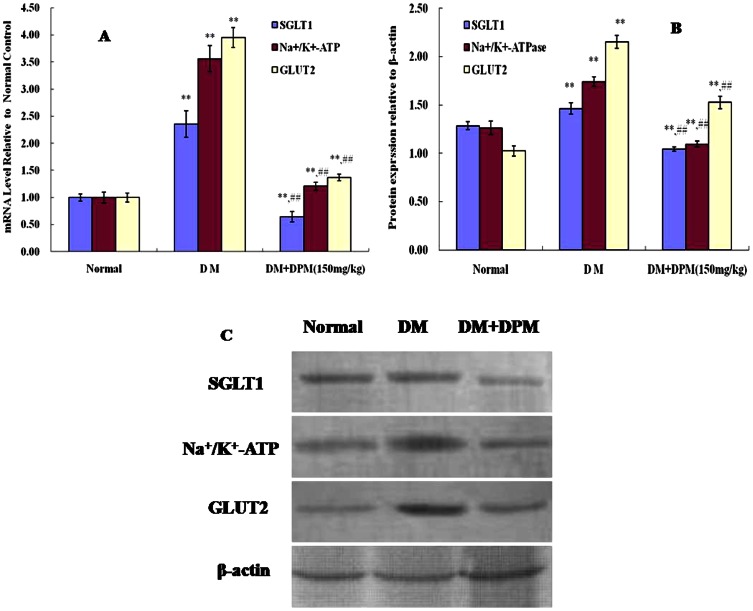
SGLT1, Na^+^/k^+^-ATPase and GLUT2 expression analysis in jejunum. **Panel** A shows RT-PCR analysis of SGLT1, Na^+^/k^+^-ATPase and GLUT2 mRNA expression. Panel B and C show western blot analysis of SGLT1, Na^+^/k^+^-ATPase and GLUT2 protein expression in normal, diabetic mice (DM) and diabetic mice treated with DPM (DM+DPM). Density values were normalized to β-actin levels. Data represent mean ±SD from three animals in each group *P<0.05, **P<0.01vs. normal; #P<0.05, ##P<0.01vs. DM.

To further explore the inhibitory effect of DPM on glucose absorption, we examined the expression of SGLT1, Na^+^/K^+^-ATP and GLUT2 in the jejunum by RT-PCR and Western blot. Levels of the mRNA (Fig. 4A) and the corresponding proteins (Fig. 4B, C) of SGLT1, Na^+^/ K^+^-ATP and GLUT2 were found to be significantly up-regulated in diabetic mice, which is consistent with previous reports [3], [4], [19]. DPM (150 mg/kg) treatment markedly suppressed expression of these proteins (P<0.01), further supporting our conclusion that DPM can inhibit glucose absorption.

### DPM accelerates hepatic glucose metabolism

**Figure 5:**
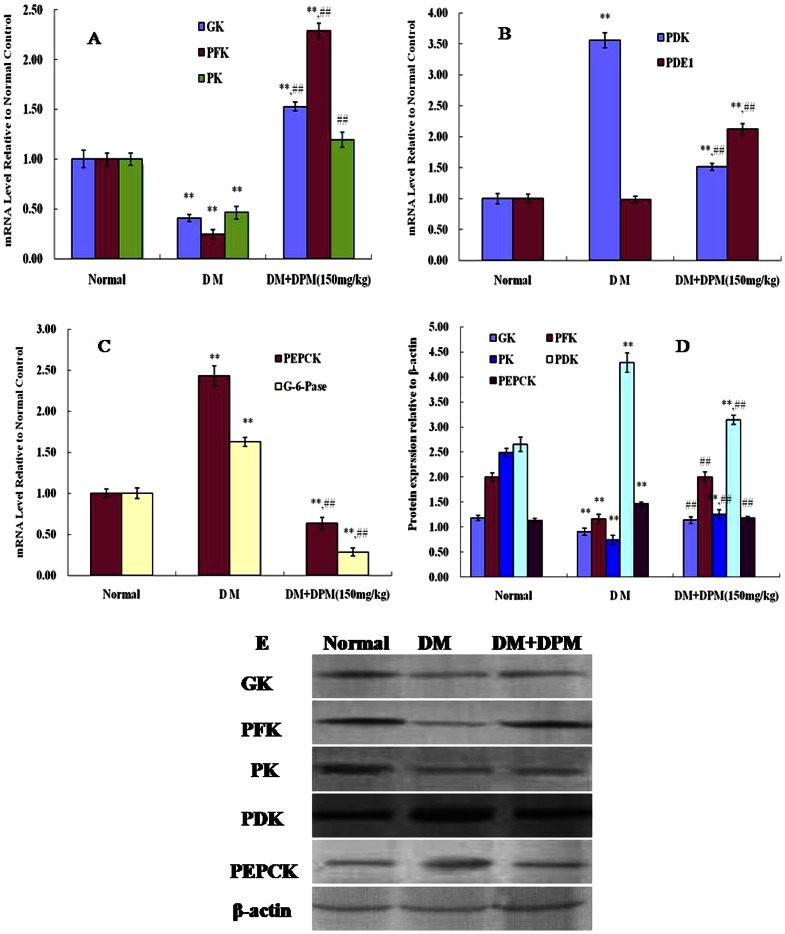
GK, PFK, PK, PDK, PDE1, PEPCK and G-6-Pase expression analysis in the liver. Panel A, B, C show RT-PCR analysis of mRNA expression in different groups. Panel D and E show western blot analysis of protein expression in normal, diabetic mice (DM) and diabetic mice treated with DPM (DM+DPM). Density values were normalized to β-actin levels. Data represent mean ±SD from three animals in each group *P<0.05, **P<0.01vs. normal; #P<0.05, ##P<0.01vs. DM.

The liver is mainly responsible for maintaining normal concentration of blood glucose by carrying out glycolysis and gluconeogenesis [27]. To assess whether DPM is involved in modulating key hepatic enzymes for glucose metabolism, the activities of GK, PFK and PK in the liver were examined. Markedly diminished activities of GK, PFK and PK were observed (P<0.01) in STZ-induced diabetic mice (Table 2). Administration of DPM (150 mg/kg) for 90 days resulted in significant increase in the activities of these glycolysis enzymes (Table 2) and corresponding increase in the expression of GK, PFK and PK mRNA as well as the respective proteins were detected in the liver (Fig. 5A, D, E).

**Table 2:** Effect of DPM on hepatic glucose metabolism enzymes in diabetic mice. (Mean ±S.D., n = 10).

Groups	GK(U/mg prot)	PFK(U/mg prot)	PK(U/mg prot)	PEPCK(U/mg prot)	G-6-Pase(U/mg prot)
Normal control	0.77±0.22	145.68±6.58	35.67±5.92	12.36±1.25	777.05±18.97
Diabetic control(DM)	0.36±0.15^**^	85.65±7.75^**^	15.53±3.12^**^	19.27±1.58^**^	1808.31±46.64^**^
Diabetic+DPM(150 mg/kg)	0.65±0.17^##^	205.56±10.87^**,##^	27.92±4.74^**,##^	15.32±0.95^*,##^	1014.07±31.99^**,##^

Data are expressed as mean ± S.D., n = 10. *P<0.05, **P<0.01, compared with normal control; # *p*<0.05, ## *p*<0.01 compared with diabetic control.

Pyruvate decarboxylase E1 (PDE1) is an important enzyme of the pyruvate dehyrogenase complex (PDC) that catalyzes the rate-limiting step of pyruvate to acetyl CoA conversion in glycolysis [28], which is inversely modulated by the PDC kinase (PDK) family [29]. DPM markedly increased PDE1 mRNA levels in diabetic mice (Fig. 5B). Conversely,PDK mRNA (Fig. 5B) and protein expression (Fig. 5 D, E) were strikingly impaired by DPM. A possible explanation for this result is that DPM catalyzes PDE1 dephosphorylation resulting in activation of PDE1. Significant decrease in hepatic pyruvate concentration was detected in DPM treated mice (Table 3), which supports the conclusion that DPM increases PDE1 activity, and consequently, accelerates the pyruvate oxidative decarboxylation reaction.

**Table 3:** Levels of hepatic glycogen and pyruvate in normal and experimental groups of mice. (Mean ±S.D., n = 10).

Groups	Glycogen(mg/g wet liver)	Pyruvate(µmol/g prot)
Normal control	52.46±4.26	25.35±1.65
Diabetic control	21.17±3.65^**^	26.89±1.71
Diabetic+DPM(150 mg/kg)	49.25±5.47^##^	18.92±1.05^**,##^

Data are expressed as mean ± S.D., n = 10. *P<0.05, **P<0.01, compared with normal control;

# *p*<0.05, ## *p*<0.01 compared with diabetic control.

**Figure 6:**
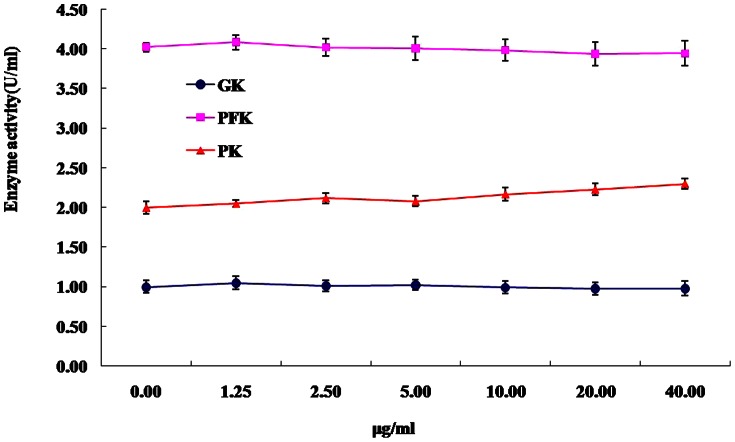
Effect of DPM on the activities of GK, PFK and PK *in vitro*. DPM at different concentrations incubated with GK, PFK, PK for 15 min before the assay. Results are expressed as mean ± SD for 3 repeats in each group.

To assess whether DPM is a direct activator of glycolysis enzymes, the effect of DPM on the enzyme activities of GK, PFK and PK were determined *in vitro* (Fig. 6). At doses ranging from 0–40 µg/ml, the activities of GK, PFK and PK were slightly altered by DPM, but no significant changes were observed. This suggests that increase in activities of these glycolysis enzymes *in vivo* did not result from direct activation by DPM, but may have been caused by increase in protein expression levels in the liver.

### DPM attenuates hepatic glucose output

To assess whether DPM plays a role in gluconeogenesis, we examined the levels of the key gluconeogenic enzymes PEPCK and G-6-Pase in the liver. Activities of these two enzymes were significantly increased in STZ-treated mice (Table 3). Oral treatment of diabetic mice with DPM resulted in marked decrease in PEPCK and G-6-Pase activities, which were, however, still higher than those in control animals. We next analyzed the gene expression levels of PEPCK and G-6-Pase in the liver of DPM treated mice. As depicted in Figure 5C, significant up-regulation in mRNA levels of the two gluconeogenic genes was detected in STZ-treated mice. In DPM treated mice, PEPCK and G-6-Pase expression levels in the liver were evidently attenuated compared to diabetic controls (Fig. 5C). Strikingly, decrease in PEPCK expression was also detected at the protein level (Fig. 5D, E). These data indicate that dephosphorylation of glucose-6-phosphate to free glucose was suppressed by DPM. Additionally, the decreased glycogen level of diabetic mice was reinstated to near normalcy in DPM treated mice (Table 3), suggesting that glucose-6-phosphate was translated into glycogen, which resulted in attenuation of hepatic glucose output upon DPM treatment.

### Histopathological studies

**Figure 7:**
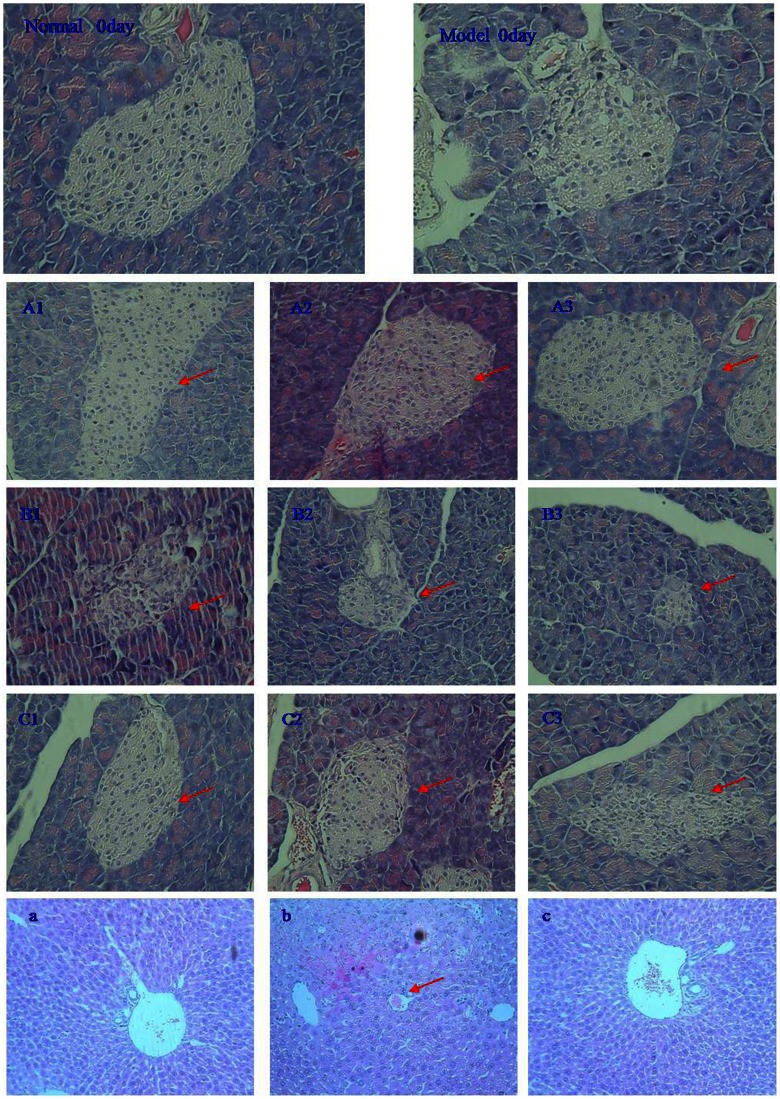
Effect of DPM on STZ-induced experimental damage to the pancreas and the liver, H&E staining, magnification, ×200. (A1, 2, 3): Control pancreas; (B1, 2, 3): Diabetic mice pancreas; (C1, 2, 3): Diabetic mice+DPM (150 mg/kg b.w.) pancreas; (a): Control liver; (b): Diabetic mice liver; (c): Diabetic mice+DPM (150 mg/kg b.w.) liver.

Seven days after STZ treatment, sections of pancreas from normal and diabetic mice were examined (H&E, ×200, Fig. 7). A clear decrease in the area occupied by β cells and structural derangement of the pancreas was observed in STZ-induced diabetic mice, indicating that the diabetic model was successfully established in terms of both hyperglycemia and partial pancreatic injury.

To further verify the therapeutic effect of DPM (150 mg/kg), sections of pancreas from normal (group I), diabetic (group VI) and DPM treated diabetic (group VII) mice were examined after DPM (150 mg/kg) treatment for 90 days (Fig. 7). Compared with pancreas from control mice (Fig. 7A), the number of β cells were significantly decreased and the structure of pancreatic islets was disordered in STZ-induced diabetic mice (Fig. 7 B1, 2, 3). These damages were reversed after 90 days of DPM treatment (Fig. 7C1, 2, 3). Repaired pancreatic β cells may contribute to improved insulin secretion, which may explain the increased serum insulin detected in DPM treated diabetic mice (Table 1). Hepatic histopathological analysis revealed hepatocyte and hepatic cord degeneration, focal necrosis and vascular congestion in the livers of STZ-induced diabetic mice (Fig. 7b). DPM (150 mg/kg) treatment significantly restored these abnormalities (Fig. 7c).

### Protective effects of DNJ and polysaccharide against STZ-induced pancreaticβ-cell damage

**Figure 8:**
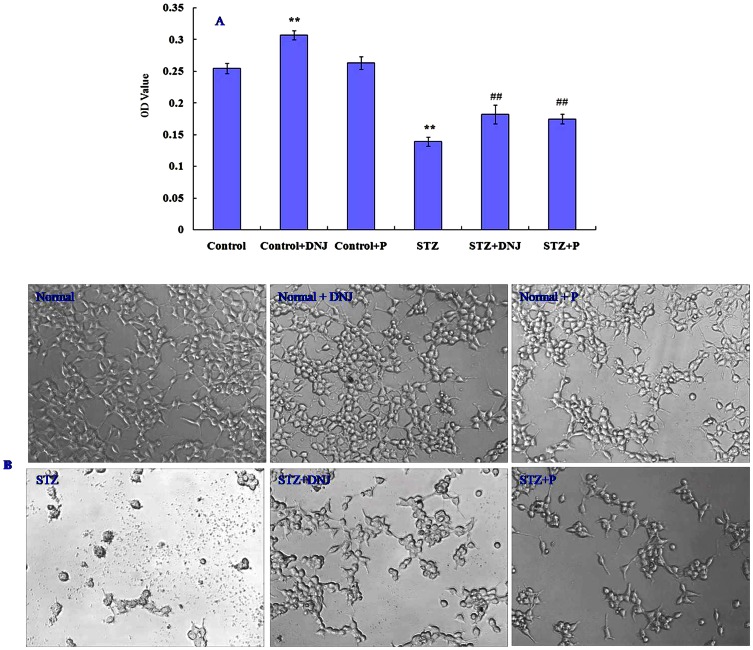
A: Effect of DNJ and polysaccharide on the pancreatic β-cells (INS-1) viability damaged by STZ. B: Effect of DNJ and polysaccharide on microscopic observation of pancreatic β-cells (INS-1). Results are expressed as mean ± SD (n = 10). *P<0.05, **P<0.01vs. Normal; #P<0.05, ##P<0.01vs. STZ model control.

Next, we investigated the protective effects of DNJ and polysaccharide against STZ-induced damage to pancreatic β-cells (INS-1). As shown in Figure 8A, cell viability was significantly lower in the STZ-treated group compared with the control group (P<0.01). Treatment with DNJ or polysaccharide (P) (200 µg/ml) for 24 h significantly reversed STZ-induced cell viability loss, as indicated by significant increase in the O.D. values (P<0.01). When observed under a microscope (Fig. 8B), normal INS-1 cells was irregular polygonal in shape and formed clusters adherent to the plate-wall. DNJ or polysaccharide treatment alone did not affect their morphology (Fig. 8B). When incubated with STZ, the INS-1 cells decreased significantly in number and exhibited pathological changes such as cell shrinkage with black spots and an overall dark appearance. Treatment with DNJ or polysaccharide (200 µg/ml) markedly restored the shape and structural integrity of the damaged cells (Fig. 8B). This indicated that the pancreatic INS-1 cells were obviously damaged by STZ, and DNJ and polysaccharide could significantly protect them from such damages. The protective effect of DNJ might result from increased proliferation of β cells (Fig. 8A), but the protective effect of polysaccharide on β cells might be related to its radical scavenging activity [15].

## Discussion

STZ-induced diabetes is a well documented model of experimental diabetes, since it shows physiological alterations similar to those observed in diabetic patients [2]. In the present study, STZ-induced diabetic mice were chosen as a model to evaluate the therapeutic potential of DPM by assessing the expression and activity of key enzymes involved in glucose absorption and metabolism. Selective destruction of pancreatic β-cells by STZ in the experimental diabetic mice model results in decreased plasma insulin level. This in turn leads to defective glucose oxidation and causes hyperglycemia [1]. Chronic hyperglycemia also leads to glucose toxicity, progressive impairment of insulin secretion and insulin resistance, similar to those observed in diabetic patients, which further worsens the control of blood glucose level [1]. Therefore, normalizing blood glucose level is an important objective in the treatment of diabetes and prevention of diabetic complications.

Elevated blood glucose levels of all diabetic mice with STZ-induced injuries of different degrees were markedly diminished after DPM treatment (150 mg/kg) for 90 days. Importantly, the decrease in blood glucose was not reversed after cessation of DPM intake for 30 days (Fig. 1), suggesting that DPM is a potent therapeutic agent against diabetes. Glycosylated hemoglobin (HbA1c) level at any given time-point reflects the mean blood glucose level over the preceding 6–8 week period. It has been shown that persistent hyperglycemia leads to non-enzymatic glycosylation of proteins [30]. In our study, diabetic mice showed higher levels of HbA1 indicating their poor glycemic control compared to control mice. Markedly decreased HbA1c levels were observed in DPM treated diabetic mice, which indicated that DPM decreased blood glucose concentration (Table 1). Histopathological examination of the pancreas showed clear decrease in the area occupied by β cells along with structural derangement in STZ-induced diabetic mice (Fig. 7B1, 2, 3). Strikingly, the decrease in pancreatic β-cells was reversed after 90 days of DPM treatment (Fig. 7C1, 2, 3). Owing to deprived pancreatic function, insulin secretion was reduced to a great extent in STZ-induced diabetic mice (Table 1). Significant increase in serum insulin levels (Table 1) was seen in DPM-treated diabetic mice, which is consistent with the reversal of pancreatic β cell loss by DPM.

To investigate the underlying mechanism for these observations, the protective effect of DNJ and polysaccharide against the damages to pancreatic β-cells (INS-1) induced by STZ toxicity was investigated. Significant decrease in the number of viable cells was detected in the STZ-treated group. In addition, INS-1 cells in the STZ-treated group exhibited marked pathological changes such as cell shrinkage with black-spots and overall dark appearance compared with the control group (P<0.01) (Fig. 8A, B), suggesting that the INS-1 cells were obviously damaged by STZ. Treatment with DNJ or polysaccharide (200 µg/ml) for 24 h significantly reversed STZ-induced cell viability loss (P<0.01) (Fig. 8A) and restored the shape and structural integrity of the damaged cells (Fig. 8B). This indicated that DNJ or polysaccharide could significantly protect the β cells from STZ-induced cell damage. Significant increase in the number of INS-1 cells in the group treated with DNJ alone indicated that the protective effect of DNJ might be from increased proliferation of β cells (Fig. 8A). The generally accepted mechanism for STZ-induced β-cell cytotoxicity is induction of oxygen free radicals [26]. We have shown in previous reports that polysaccharides in DPM can protect pancreatic islets from alloxan-induced damage by scavenging free radicals. Since polysaccharides were not found to increase the proliferation of β cells in this study, we speculate that the protective effect of polysaccharide on β cells may result from its radical scavenging activity [15]. Taken together, these results strongly support the therapeutic potential of DPM against diabetes.

Carbohydrates, which are one of the three major constituents of mammalian diet, are hydrolyzed thoroughly by digestive enzymes in the gastrointestinal tract [11]. For this reason, glycosidases were the initial therapeutic targets for treatment of diabetes. Strikingly, pretreatment of diabetic mice with DPM (150 mg/kg) significantly attenuated the hyperglycemia induced by sucrose (Fig. 3A), indicating that DPM is a potent inhibitor of intestinal sucrase. This is consistent with previous studies in which DNJ in DPM has been reported to be a potent inhibitor of intestinal glycosidases [12], [15]. After carbohydrates are hydrolyzed into monosaccharides, glucose is absorbed through the intestine by a transepithelial transport system initiated at the apical membrane by the cotransporter SGLT1 [4]. Intracellular glucose then diffuses across the basolateral membrane with the help of glucose transporter 2 (GLUT2) [4], [31]. Diabetic animals have been reported to have increased capacity for glucose absorption via changes in the BBM and BLM, specifically via enhanced activity and abundance of SGLT1 and GLUT2 [4], [19]. Therefore, SGLT1 and GLUT2 are considered potential targets of drug development for glycemic control in diabetic patients [32]. Uptake of glucose across the BBM is also mediated by Na^+^/K^+^-ATPase, which is responsible for establishing and maintaining the Na^+^ gradient required for the activity of the Na^+^/glucose cotransporter (SGLT1) [4]. Recent studies have shown that modifications of systemic glycemia in OGTT reflect the activity of the intestinal glucose transporter SGLT1 [33]. Therefore, we assessed the effect of DPM on diabetic mice subjected to OGTT. Maximal blood glucose levels were significantly lower than in the corresponding control at 30 min after glucose load (Fig. 3B), and remarkable amelioration of glucose tolerance was observed in glucose tolerance curves (Fig. 3B, D), indicating reduction of intestinal glucose transport *in vivo*. Labeled glucose uptake assay showed that ^13^C_6_-glucose level was remarkably lower in the serum, but significantly higher in the intestine in DPM treated mice than in diabetic controls (Fig. 3E, F), supporting the conclusion that DPM attenuates glucose transportation from the small intestine to the blood. In addition, consistent with previous reports [4], [5], [19], we found that the mRNA and protein levels of SGLT1, Na^+^/K^+^-ATPase and GLUT2 were up-regulated in the jejunum of STZ-induced diabetic mice (Fig. 4). Strikingly, expressions of all three proteins were decreased after DPM treatment for 90 days (Fig. 4B, C). These findings add further support to the contention that DPM can inhibit glucose absorption, making it a potent therapeutic candidate for diabetes treatment.

Liver is an important organ that plays a pivotal role in glycolysis and gluconeogenesis [27]. In glycolysis, GK, PFK and PK are the key rate limiting enzymes mediating oxidation of glucose, which catalyzes the conversion of glucose to pyruvate and results in ATP generation [9], [27]. Pyruvate decarboxylase E1 (PDE1) is an important enzyme in the pyruvate dehyrogenase complex (PDC) that catalyzes the rate-limiting step of pyruvate to acetyl CoA conversion [28], which is reversely mediated by the PDC kinases (PDK) family [29]. Many researchers have reported that GK, PFK and PK are the most sensitive indicators of the glycolytic pathway in diabetic animals. Insufficiency of these enzymes in the diabetic state can cause decreased utilization of glucose for energy production [34], [35]. In the current study, we found marked increase in the activities of these glycolysis enzymes in diabetic mice treated with DPM (150 mg/kg) for 90 days (Table 2). Additionally, mRNA and protein expression levels of hepatic GK, PFK and PK were also strikingly increased in DPM pretreated mice compared to diabetic controls (Fig. 5 A, D, E). Increase in activity of glucose metabolism enzymes in the liver may result from either increase in enzyme activity, or up-regulation of expression of the enzymes. *In vitro* experiments revealed that DPM had no effect on the activity of GK, PFK and PK, which suggests that the increased activities of these glycolysis enzymes observed *in vivo* resulted from increase in protein expression. Partial deficiency of insulin in diabetic mice has been shown to attenuate the activities of these glycolysis enzymes [36], so the enhanced activity of these glycolysis enzymes may also be attributable in part to increased serum insulin resulting from improved pancreatic β cell health upon long term treatment with DPM (150 mg/kg) (Table. 1). Although our results show that STZ treatment does not result in significant down-regulation of PDE1 gene expression in the liver, DPM markedly increased PDE1 mRNA levels in diabetic mice (Fig. 4B). Conversely, attenuation of PDK mRNA and protein expression (Fig. 4B, D and E) was observed in DPM pretreated mice. The decrease in PDK might induce dephosphorylation and consequent activation PDE1. Furthermore, significant decrease of hepatic pyruvate concentration was detected in DPM treated mice (Table 3), suggesting that the pyruvate oxidative decarboxylation reaction was accelerated. Taken together, these results indicate that DPM accelerates hepatic glucose metabolism.

In gluconeogenesis, PEPCK is involved in the synthesis of glucose-6-phosphate from non-carbohydrate precursors, and G-6-Pase catalyzes dephosphorylation of glucose-6-phosphate to provide free glucose to other organs during diabetes, prolonged fasting, or starvation [37]. Enhanced activities and expressions of PEPCK and G-6-Pase in the liver have been observed in most diabetic models, and are thought to contribute to increased hepatic glucose output in this disease [38]. DPM (150 mg/kg) treatment of diabetic mice resulted in marked decrease of PEPCK and G-6-Pase activities (Table 3) and their mRNA levels in the liver (Fig. 5C). Decrease in protein expression of PEPCK was also detected in the DPM treated group compared to diabetic controls (Fig. 5D, E). These results are consistent with our previous studies [15] in which expression of PEPCK and G-6-Pase was found to be inhibited in DPM treated mice with alloxan-induced diabetes. Considering the observed decrease in blood glucose level, these data indicate that DPM suppresses dephosphorylation of glucose-6-phosphate to form free glucose. Glycogen is the primary intracellular storable form of glucose and its levels in various tissues, especially in the liver, kidney and skeletal muscles, directly reflect insulin activity, which regulates glycogen deposition by stimulating glycogen synthase and inhibiting glycogen phosphorylase [39]. Increased serum insulin (Table 1) and reinstatement of glycogen levels (Table 3) in diabetic mice with DPM treatment suggests that DPM facilitates conversion of glucose-6-phosphate to glycogen, which then results in attenuation of hepatic glucose output. In addition, we also found that the abnormal organization of hepatocytes in STZ-induced diabetic mice was ameliorated after 90 days of DPM treatment (Fig. 7C). However, additional studies are needed to further understand the protective role of DPM on the liver.

In summary, we have shown that DPM has therapeutic effects against diabetes via multiple pathways. It inhibits glucose absorption in the small intestine by attenuating the expression of transepithelial glucose transport proteins, keeps blood glucose level stable by directly regulating the expression of enzymes involved in glycolysis and gluconeogenesis in the liver, and restores damaged pancreas to normalcy by scavenging free radicals and increasing β-cell proliferation. Therefore, DPM may provide a valuable therapeutic option against diabetes.
